# A Meiotic Tapas Menu

**DOI:** 10.1371/journal.pgen.0020019

**Published:** 2006-02-24

**Authors:** Gregory P Copenhaver

**Affiliations:** Stowers Institute for Medical Research, United States of America

Like Muhammad Ali's 1974 “Rumble in the Jungle,” this past fall (September 13–18), the 7th European Meiosis Meeting held in San Lorenzo de El Escorial brought us “Meiosis in Madrid.” This is a sister conference to the Gordon Meiosis series held every other year in the United States, and is an international small-format forum for sharing hot, new results, with an emphasis on unpublished work. At the conference, 21 countries were represented by 167 participants, with a nearly equal split between male and female scientists. The aim of the meeting was to present and promote in-depth discussions about all aspects of meiotic chromosome dynamics, recombination, and segregation. Rather than providing a comprehensive description of the meeting abstracts, this report will briefly describe meeting highlights, guided in large part by feedback from session chairs.

## Beginnings and Current Questions

**Figure 1:**
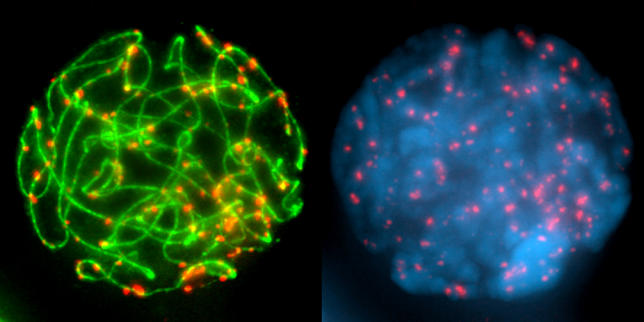
Synapsis and Recombination Can Be Visualized on Chromosomes from Grasshopper *(Eyprepocnemis plorans)* Spermatocytes Immunodetection of the cohesin subunit SMC3 (green) and the histone variant γ-H2AX (red) on DAPI stained (blue) pachytene chromosomes. (Image: Julio Sánchez Rufas, Universidad Autónoma de Madrid)

In an unusual but enlightening departure from the emphasis on “hot off the press” results, Gareth Jones (University of Birmingham, United Kingdom) kicked the meeting off with a retrospective keynote address that stretched back to the roots of meiotic investigation in the mid-19th century. Meiosis is an intricate process that requires the movement and rearrangement of large macromolecules. Despite its complexities, meiosis can be thought of as comprising three essential steps. Mom and dad's chromosomes (homologs) have to find one another. They have to form stabilizing connections—typically by breaking and rejoining in a way that swaps DNA between the homologs in a process known as crossing over. Finally, the chromosomes have to separate and move to daughter cells. Jones's review highlighted the fact that some of our most powerful insights and lasting impressions of meiosis have come from visualizing chromosomes by cytogenetics. It was, therefore, exciting to see that several of the Spanish labs hosting the conference are continuing to push the boundaries of cytogenetics with compelling results (Figure 1).

Even though meiosis has been the object of intense scientific scrutiny for well over a century, several important questions still remain unanswered. How do homologous chromosomes, particularly in species with significant genomic redundancy, accurately recognize one another? Once pairing is achieved, a proteinaceous structure called the synaptonemal complex (SC) forms, creating an intimate associate between the pairs [1]. How the SC is restricted to properly paired homologs and what it does once it is established is not well understood. It is clear that, in most species, crossovers (COs) between each pair of homologs are a prerequisite for proper chromosome segregation. Conversely, each CO represents the opportunity for a catastrophic error resulting in inappropriate chromosomal rearrangements. As a result, COs are thought to be tightly regulated by largely unknown mechanisms. Indeed, we have yet to elucidate all of the molecular players that mediate recombination. After pairing and recombination, the four chromatids present at meiosis interact by way of their centromeres with the cell's conveyor belt—the spindle apparatus—to facilitate movement toward the poles. In the first meiotic division, sister chromatids orient themselves toward the same pole, with homologous pairs oriented to opposite poles. Subsequently, in preparation for the second meiotic division, sister chromatids become oriented to opposite poles. The cellular machinery that establishes and then changes these topological orientations, as well as those that effect chromosome movement on the spindle, remains for the most part enigmatic.

## The Dynamics of Chromosome Pairing

As described above, homolog recognition, pairing, and SC formation are critical meiotic processes. Adela Calvente (Universidad Autónoma de Madrid, Spain) described results using a grasshopper species *(Stethophyma grossum)* with fully, partially, and unsynapsed chromosomes to investigate the relationship between meiotic recombination and the formation of the SC. Using immunolocalization, she showed that when the distribution of recombination events is restricted to specific domains, chromosomal synapsis is coordinately restricted. In most organisms prior to pairing, chromosomes form a cluster called a bouquet with their telomeres grouped at the nuclear envelope [2]. Tomas Naranjo (Universidad Complutense, Spain) also used cytogenetics in hexaploid wheat *(Triticum aestivum)* to show that centromere and, more importantly, telomere clustering during bouquet formation help facilitate homolog recognition. Abby Dernburg (University of California Berkeley, US) showed that Caenorhabditis elegans, which lacks a bouquet, uses an alternate system wherein specialized chromosomal domains known as pairing centers are bound by specific proteins that then associate with protein complexes (or “patches”) in the nuclear envelope, thereby facilitating chromosome recognition and pairing.

## Recombination Double-Strand Breaks

Meiotic recombination is now thought to begin with the formation of double-strand breaks (DSBs) in DNA through the action of a topoisomerase-like protein called Spo11. How these breaks are then repaired has been a hotly debated focus in the field. In a process initially described by Szostak et al. [3], the 5′ ends of the break are resected to leave 3′ single-stranded tails. One of these ends invades a homologous chromatid and primes new DNA synthesis. The other end then captures the resultant DNA loop and uses it as a template for new synthesis. Joining the free ends generates a recombination intermediate linked by what is known as a “double Holliday junction.” This intermediate can be resolved to produce a CO or a noncrossover (NCO). This model superseded previous models in which single-stranded nicks were thought to be the initiating event.

Spo11, after cleaving the DNA, remains covalently linked to the 5′ ends of the DSB until it is enzymatically removed and single-stranded 3′ ends are formed during resection [4]. Scott Keeney (Memorial Sloan-Kettering Cancer Center, US) showed evidence that Spo11 is removed from the 5′ ends by endonucleolytic processing events that result in the release of Spo11 still attached to oligonucleotides of two defined sizes. This result rules out a previous model asserting that Spo11 is removed from DSB ends by reversing the transesterification process that initially attached it. Importantly, the process creates an asymmetry at the ends of DSBs, and thus may have implications for recombination, which is also thought to proceed via an asymmetric mechanism.

Gerry Smith (Fred Hutchinson Cancer Research Center, US) presented data showing that he can detect recombination DNA intermediates (joint molecules) in a *mus81* mutant Schizosaccharomyces pombe strain. These molecules have been created by the action of Rec12 (*S*. *pombe*'s Spo11). The persistence of these intermediates supports the idea that Mus81 is a joint molecule resolvase. This evidence is exciting since there are few, if any, other examples of stabilized recombination intermediates outside of Saccharomyces cerevisiae. Smith also hypothesized that the joint molecules he detects represent single Holliday junctions, suggesting that *S*. *pombe* utilizes a recombination pathway that is different than the canonical DSB repair pathway modeled in *S*. *cerevisiae*.

Recombination can occur between sister or nonsister chromatids, but in most organisms, nonsister interactions mediated by a protein called Dmc1 are favored during meiosis. Nancy Hollingsworth (State University of New York at Stony Brook, US) showed that a kinase called Mek1 is required to prevent sister chromatid repair in *dmc1* mutants. This argues against previous models that suggested that interhomolog bias is not due to a suppression of sister chromatid recombination, but, instead, is due to the active promotion of interhomolog events.

Monique Zetka (McGill University, Canada) showed evidence that in *C*. *elegans,* Htp-3 (a chromosome axis component) is required for the formation of meiotic DSBs—a surprising result for a structural protein. She suggests that Htp-3 may play a role similar to Red1 [5]—the Spo11 complex may require contact with axis proteins in order to form a stable DSB. Alternatively, localizing Htp-3 to the meiotic axis may signal the formation of a meiotic chromosome structure recognized by the cell as being permissive for DSB formation.

## Recombination Early Pathway

Repairing DSBs can result in either a CO or an NCO. Some researchers now believe that NCOs come from an alternative recombination pathway, termed synthesis-dependent strand annealing [6]. In this pathway, the invading strand dissociates from the template after some synthesis. The newly synthesized region can anneal to the other resected end of the break. Additional fill-in synthesis and ligation results in an NCO. The decision to repair a DSB as either a CO or an NCO is now thought to occur early [7].

Mutants in the Zip3 protein in *S*. *cerevisiae* are required for both the CO pathway and for the initiation of the SC. Ting-Fang Wang (University of California Berkeley, US) presented evidence suggesting that Zip3 is an Smt3 E3 ligase, and that Zip1 (a structural protein that makes up the central region of the SC) binds to Smt3-conjugated products along the meiotic chromosomes to form the SC. Gillian Hooker (Howard Hughes Medical Institute, US) also showed that Smt3 localizes to synapsed regions of yeast meiotic chromosomes. In *zip1* cells, which lack SC but still show punctate association between homolog axes, Smt3 localized to sites of axis association.

Several mutants, including *com1*/*sae2*Δ, *rad50S,* and *mre11S,* are defective in repairing Spo11-induced DSBs, and, instead, accumulate 5′ ends covalently bound by Spo11. Franz Klein (University of Vienna, Austria) examined whether *com1*/*sae2*Δ*, rad50S,* and *mre11S* are defective in processing substrates containing hairpins, as suggested by previous observations. They ligated hairpin-forming oligonucleotides to the ends of a linearized integrative plasmid, and showed that these integrated into the genome at normal levels in wild-type cells, but only a few transformants could be recovered from the mutants. When analyzed, most of the residual transformants in *com1*/*sae2*Δ cells did not have distal plasmid sequences integrated, suggesting that the event did not involve the hairpin-capped ends.

Neil Hunter (University of California Davis, US) and Michael Lichten (National Cancer Institute, US) presented data on *S*. *cerevisiae SGS1* suggesting that the Sgs1 helicase does not markedly affect CO frequencies in wild-type yeast (SK1). This stands in contrast to previous reports of a different strain that had a significant increase in COs, highlighting, once again, the importance of strain differences in these experiments. Nonetheless, two salient points emerged. In mutants that are specifically CO defective due to an absence of either biochemical activities or SC components, Sgs1 helicase has a major anti-CO activity. Deletion of the helicase domain substantially restores COs to all of these mutants, in some cases to wild-type levels. Also, *sgs1* mutants show significant spore inviability (about 20%–30% of wild type). Beth Rockmill (Yale University, US) showed that this spore inviability is at least partially suppressed by mutants that reduce the activity of Spo11, which reduces COs. She also reported, in *sgs1* mutants, elevated COs in the vicinity of the centromere on Chromosome III, and showed that chromatids with these centromere-adjacent COs are at increased risk for premature sister chromatid separation. In fact, she calculates that 40% of the COs that occur within 1 kilobases of the centromere show premature sister chromatid separation. This is not specific to *sgs1* mutants—the same is true in wild type; there are simply more COs in *sgs1*. This is an important finding since it shows that COs near the centromere compromise proper chromosome segregation, thus providing a reason for why centromeres are kept cold for recombination in a wide variety of organisms.

## Regulation of Recombination

In most organisms, genetic exchanges are not distributed randomly among populations of cells or along individual chromosomes. Instead, cells typically regulate the number and placement of COs in a way that ensures that each chromosome experiences at least one (thus stabilizing homolog pairing), and also distributes them in a quasi-uniform pattern using a poorly understood phenomenon known as CO interference.

In addition to regulating the number and placement of recombination events, the cell must also ensure their fidelity. Chris Franklin (University of Birmingham, UK) showed that the Zyp1 protein in Arabidopsis thaliana is critical for ensuring that COs only occur between homologous chromosomes. Plants that carry mutant alleles of the two redundant copies of this gene experience nonhomologous crossing over and are defective in forming SC. Gregory Copenhaver (University of North Carolina at Chapel Hill, US), also working in *A*. *thaliana*, showed evidence that COs in this organism fall into two classes—those sensitive to CO interference and those that are insensitive. In addition, he showed that the ratio of these types of COs varies from chromosome to chromosome in a way that may reflect chromosomal architecture.

Early steps in meiotic recombination provide critical regulatory points for controlling the frequency, distribution, and type of recombination events that occur. In *S*. *cerevisiae,* a recombinase called Dmc1 mediates homology searching, strand invasion, and strand exchange. Regulating where Dmc1 can act is therefore important. Doug Bishop (The University of Chicago, US) provided evidence that Tid1/Rdh54 and its paralog, Rad54, act to prevent sequestration of Dmc1 at nonspecific sites on chromatin, thereby making it possible for the recombinase to assemble at DSB sites. This suggests that disruption of recombinase–double-stranded DNA interaction is an important energy-requiring step in recombination.

Recently, proteins that serve as accessory factors to Dmc1 have been identified. Several lines of evidence, including new work presented on the *A*. *thaliana Mnd1* gene by Peter Schloegelhofer (University of Vienna, Austria), show that one of these accessory factors, the Hop2/Mnd1 heterodimer, is conserved across many, but not all, species. Although the molecular mechanism of Hop2/Mnd1 stimulation of Dmc1 is not yet fully understood, Dan Camerini-Otero (National Institutes of Health, US) showed that neither the intrinsic strand invasion activity of the mouse Hop2 protein nor the DNA binding by Hop2/Mnd1 are required for the stimulation of Dmc1 by the Hop2/Mnd1 heterodimer. Furthermore, the mammalian Hop2/Mnd1 complex stimulates DNA binding by Dmc1 up to 30-fold.

Alec Jeffreys (University of Leicester, UK) discussed mammalian meiotic recombination hotspots identified initially by linkage disequilibrium studies followed by molecular sperm-typing studies. He found that linkage disequilibrium hotspots are different in humans and chimps, implying that they are short-lived over evolutionary time. Several of the human hotspots are polymorphic, thus enabling transmission studies that show that the recombination cold alleles are overtransmitted and reciprocal COs are displaced relative to each other, as one might expect for heteroduplex DNA tracts. Also, at two loci where a single nucleotide polymorphism is associated with a hotspot, the ancestral allele is the active one. Finally, one hotspot appears to be new since it is present in only three of 23 men, and all three men appear to be hotspot heterozygotes.

Bernard de Massey (Institute of Human Genetics, France) described data from scoring recombination at the molecular level during mouse spermatogenesis. He scored both COs and NCOs. By examining testes 11–21 days postpartum, his group was able to examine timing and genetic dependence of COs and NCOs. Their results show that NCOs appear a little before COs. Additionally, they showed that in *mlh1* mice, COs are reduced 10–20 fold while NCOs are unaffected.

## Chromosome Segregation

Pictures of spindle microtubules emanating from the centrosome during meiosis are so familiar that they are textbook classics. Ironically, many organisms, including *Drosophila,* lack a centrosome and, instead, build anastral spindles. Kim McKim (University of New Jersey, US) discussed insights into *Drosophila* spindle assembly based on analysis of Subito, a kinesin-like protein. In wild-type cells, Subito associates with the meiotic central spindle, while in mutant cells, the central spindle is defective, spindles are tri or monopolar, and homologous chromosomes do not properly separate at meiosis I. These observations suggest that Subito is required for central spindle assembly or maintenance.

In addition to lacking centrosomes, *Drosophila* also differ from other organisms because they can appropriately segregate their chromosomes in the absence of crossing over (in males, and in the small fourth chromosome and in sex chromosomes in females). R. Scott Hawley (Stowers Institute for Medical Research, US) described surprising new evidence that achaismate chromosomes in *Drosophila* oocytes are, in fact, connected to one another by what appear to be heterochromatic DNA. Hawley hypothesizes that these linkages are recombination intermediates caused by stalled replication forks, and that they can be resolved in a way that does not create a CO but does allow information exchange.

## Summary

As is customary in Spain, the intellectual menu at this meeting was both rich and varied. Most importantly, the meeting highlighted the spirit of international scientific cooperation and an open exchange of new ideas accompanied by critical but constructive discussion.

## Abbreviations

CO: crossover
DSB: double-strand break
NCO: noncrossover
SC: synaptonemal complex
